# Editorial

**Published:** 1891-08

**Authors:** 


### Editorial.

### PRESCRIPTION WRITING.

For importance, and as evidence of scholastic attainments, few things surpass a prescription.

Why the medical profession should so alarmingly ignore the grammatical laws of prescription writing, or persist in ignorance of the same, is, indeed, an enigma.

If a man know not his Latin grammar, we feel sure that any one worthy his degree of Doctor in Medicine can, in a few weeks, learn from any work on the subject of prescription writing to at least write a decent prescription.

Generally speaking, in a Latin prescription, the genitive and accusative cases are the only ones required, and though there be exceptions to each declension, they are few in number, and can with a little thought and study soon be committed to memory.

How humiliating to hear a pharmacist deride an order sent by some man with a large practice to be dispensed. How embarrassing to hand a poorly written and incorrect prescription to a patient of classic attainments, and to see on his countenance an expression of surprise and wonder as he glances over the same and notes many egregious errors.

Then further, ignorance is so often accompanied by fulsome self-conceit. To see a man write a prescription in incorrect Latin combined with equally bad English, and abbreviations both incorrect and confusing, is indeed a mortifying position to one who regards his profession with the proper respect. Now, the remedy for such evils is for our colleges and medical journals to inculcate the principles of classic thought and ambition, or to so dignify an English prescription that those who cannot, or will not, forbear the pleasure of writing in Latin, when "they know not what they do," can retain their personal dignity by using simple and pure English.

DOCTOR IN MEDICINA.

INTER-CONTINENTAL AMERICAN MEDICAL CONGRESS.—The object of this is to have a meeting of the medical men of the Western Hemisphere assembled in one congress. A committee was nominated at the last meeting of the American Medical Association. The following officers were elected: Charles A. L. Reed, Cincinnati, chairman; J. W. Carhart, Lampasas, Texas, secretary; I. N. Love, St. Louis, treasurer. An adjourned meeting will be held at St. Louis, October 14, 1891, when a constitution will be presented and the time and place of meeting of the congress decided, as well as the election of permanent officers.

---

Dr. G. Frank Lydston, of Chicago, has been elected Professor of Genito-Urinary Disease in the College of Physicians and Surgeons of Chicago. A better selection for the chair could not have been made, for the Doctor stands at the head in his branch.

---

### DR. J. B. S. HOLMES.

Dr. J. B. S. Holmes, of Rome, Ga., was in the city a few days ago and paid us a visit. The doctor is building a private sanitarium for the treatment of diseases of women. We will have something more to say about this sanitarium later.

---

Dr. Bransford Lewis will start shortly a new journal in St. Louis, and offers special inducements to subscribers, which will be found in our "ads." Judging by the Dr.'s letters, of which we have published several, his new departure should be a success.

---

THE WOMAN'S MEDICAL COLLEGE OF GEORGIA opens October 1st, with prospects of a large class. The ladies of the Board of Trustees, with Mrs. Gov. Northen as President, and the Faculty, with Dr. A. G. Thomas as President and Dr. J. W. Stone as Dean, are bringing this institution to great usefulness to our Southern ladies. One-half reduction of lecture fees is granted to wives and daughters of physicians, clergymen and Confederate veterans. For particulars, address Woman's Medical College, Atlanta, Ga., Box 215.

